# Die neue WHO-Klassifikation der Kiefertumoren

**DOI:** 10.1007/s00292-023-01195-4

**Published:** 2023-05-13

**Authors:** Simon Haefliger, Daniel Baumhoer

**Affiliations:** Knochentumor-Referenzzentrum am Institut für Pathologie, Universitätsspital Basel, Universität Basel, Schönbeinstr. 40, 4031 Basel, Schweiz

**Keywords:** Kraniofaziale fibröse Dysplasie, Kopf- und Halstumoren, Odontogene Tumore, Rhabdomyosarkom, Weltgesundheitsorganisation, Craniofacial fibrous dysplasia, Head and neck neoplasms, Odontogenic tumours, Rhabdomyosarcoma, World Health Organization

## Abstract

Odontogene Tumoren und Kiefertumoren umfassen ein breites Spektrum an Läsionen, darunter Neoplasien, hamartomatöse Veränderungen und Entwicklungsstörungen. Seit Anfang 2022 ist eine Beta-Version der 5. Auflage der WHO-Klassifikation für Kopf-Hals-Tumoren online verfügbar, die voraussichtlich Mitte 2023 auch als Printversion erscheinen wird. Konzeptionell hat sich im Vergleich zur 4. Auflage nicht viel verändert. Die Reihenfolge der Läsionen ist stringenter nach Dignität geordnet und die gleichen Tumoren werden nicht mehr in verschiedenen Kapiteln lokalisationsabhängig mehrfach beschrieben. Diagnostische Kriterien werden neu in „essential“ und „desirable“ zusammengefasst. Zusätzlich zu den klinischen Aspekten wird nun auch die Bildgebung miteinbezogen und die Klassifikation damit interdisziplinär. Vereinzelt sind auch neue Läsionen erstmals aufgeführt. Dieser Artikel gibt einen Überblick über die Neuerungen der neuen Klassifikation mit einem speziellen Fokus auf die Einteilung der fibroossären Kieferläsionen.

## Abstract (en)

Maxillofacial tumours cover a broad spectrum of lesions, including neoplasms, hamartomatous changes and developmental disorders. Since the beginning of 2022, a beta version of the 5th edition of the WHO classification for head and neck tumours has been available online, and a print version is expected to be published in mid-2023. From a conceptual point of view, little has been changed compared to the 4th edition; the sort order of lesions is more rigorously arranged according to benign and malignant behaviour and identical tumour types are no longer described redundantly in different chapters depending on their location. The diagnostic criteria are now summarized as “essential” and “desirable”, and in addition to the clinical features, imaging is now also incorporated, providing an interdisciplinary approach to the classification. A few new entities are included for the first time. This article gives an overview of the main changes introduced in the new WHO classification with a special emphasis on fibro-osseous lesions of the craniofacial skeleton.

Die voraussichtlich 2023 erscheinende 5. Auflage der WHO-Klassifikation für Kopf‑/Hals-Tumoren wird 6 Jahre nach der Vorgängerversion publiziert. Dieser relative kurze Abstand ist in erster Linie darauf zurückzuführen, dass die WHO die 5. Auflage sämtlicher Klassifikationen zunächst abschließt, bevor die ersten Bände in der 6. Auflage erscheinen. Auch gesamthaft werden die Abstände zwischen den Auflagen kürzer, da der molekulargenetische Fortschritt sonst nicht mehr adäquat abgebildet werden kann.

Die 5. Auflage der Kopf-Hals-Tumorklassifikation enthält bei Kiefertumoren nur wenige neue oder erstmalig aufgenommene Läsionen. Sämtliche Kapitel wurden aber vollständig aktualisiert und größtenteils neu verfasst. Zusätzlich wird nun verstärkt einem zunehmend interdisziplinärem Ansatz in der Diagnostik Rechnung getragen, indem die einzelnen Kapitel auch eine Beschreibung der Bildgebung enthalten. Die Reihenfolge der verschiedenen Läsionen innerhalb eines Kapitels folgt stringent der Dignität (beginnend mit den gutartigen Tumoren). Zudem werden nun essenzielle und wünschenswerte („desirable“) diagnostische Kriterien separat aufgeführt.

Das Kapitel der odontogenen Tumoren und maxillofazialen Knochentumoren gliedert sich in Kieferzysten, odontogene Tumoren (benigne epitheliale, benigne gemischte, benigne mesenchymale und maligne Tumoren), riesenzellhaltige Läsionen und Knochenzysten sowie Knochen- und Knorpeltumoren (fibroossäre Läsionen, benigne und maligne Tumoren). Die traditionelle Einteilung der benignen odontogenen Tumoren in epitheliale, gemischte und mesenchymale Tumore behält die neue Auflage unverändert bei. Zu den erstmaligen aufgeführten Entitäten in der Gruppe odontogener Tumoren und Zysten gehören die postoperative Flimmerepithelzyste („post-surgical ciliated cyst“) und das adenoide Ameloblastom. In der Gruppe der fibroossären Tumoren und Dysplasien werden neu die segmentale odontomaxilläre Dysplasie (SOD) und die floride familiäre zementoossäre Dysplasie (FFZOD) eingeführt. Als einzige neue Entität bei den Knochentumoren wird das Rhabdomyosarkom mit *TFCP2*-Rearrangement aufgenommen. Einige hier nur exzeptionell selten vorkommende Entitäten wurden zudem aus dem Kapitel der Kiefertumoren gestrichen. Hierzu zählen der melanotische neuroektodermale Tumor des Kleinkindesalters, das Osteoidosteom und das solitäre Plasmozytom.

In den folgenden Abschnitten werden die wichtigsten Änderungen genauer dargestellt. Besonders im Fokus sind hierbei die häufiger vorkommenden fibroossären Läsionen. Einen Überblick über wesentliche Neuerungen der neuen Ausgabe der WHO-Klassifikation sowie eine kurze Zusammenfassung der neu eingeführten Entitäten geben Tab. 1 und 2.

**Table 1:** 

Kategorie	Entität	Änderungen (Klassifikation und/oder Pathogenese)
*Odontogene Zysten*	Postoperative Flimmerepithelzyste	Erstmals aufgeführt (siehe auch Tab. 2)
	Kalzifizierende odontogene Zyste	Vereinfachung der diagnostischen Kriterien, Beschreibung von Mutationen im Gen *CTNNB1*-Mutationen
*Odontogene Tumoren*	Ameloblastom	Neue Nomenklatur: konventionell anstatt solide/multizystisch
	Adenoides Ameloblastom	Erstmals aufgeführt (siehe auch Tab. 2)
	Kalzifizierender epithelialer odontogener Tumor	Einführung von 3 histopathologischen Subtypen (klarzellig, zystisch/mikrozystisch und nichtkalzifizierend/Langerhans-Zell-reich)
	Fibromyxom	Neue Nomenklatur: Fibromyxom anstelle von Myxofibrom
	Zementoossifizierendes Fibrom	Neue Klassifikation als benigner, mesenchymaler, odontogener Tumor (zuvor bei den fibroossären Läsionen)
*Riesenzellhaltige Läsionen und Knochenzysten*	Zentrales und peripheres Riesenzellgranulom	Beschreibung von pathogenen Hotspotmutationen in den *KRAS-, FGFR1-* und *TRPV4-*Genen
	Aneurysmatische Knochenzyste	Neue Nomenklatur: primär vs AKZ-artige Veränderungen oder zystische hämorrhagische Degeneration (nicht mehr sekundäre AKZ)
*Fibroossäre Läsionen des Kiefers*	Floride familiäre zementoossäre Dysplasie	Erstmals aufgeführt (siehe auch Tab. 2)
	Segmentale odontomaxilläre Dysplasie	Erstmals aufgeführt (siehe auch Tab. 2)
	Psammomatoides ossifizierendes Fibrom	Neue Nomenklatur: Der zuvor verwendete Begriff „juvenil“ wurde aufgegeben
*Benigne Knochen- und Knorpeltumoren*	Zementoblastom	Beschreibung von *FOS*-Rearrangements
*Maligne Knochen- und Knorpeltumoren*	Rhabdomyosarkom mit *TFCP2*-Rearrangement	Erstmals aufgeführt (siehe auch Tab. 2)
	Chondrosarkome	Neue Nomenklatur: Beschreibung als „Chondrosarkom Tumorfamilie“ (= konventionell, periostal, dedifferenziert und klarzellig) vs mesenchymale Chondrosarkome (bleiben separat)

**Table 2:** 

Entität	Epidemiologie und Besonderheiten	Wesentliche diagnostische Kriterien
*Postoperative Flimmerepithelzyste*	5. bis 6. Dekade, keine Geschlechterpräferenz	St. n. Voreingriff
	V. a. hintere Maxilla	Scharf begrenzte Osteolyse
		Von respiratorischem Epithel ausgekleidete Zyste
*Adenoides Ameloblastom*	Breite Altersverteilung (peak in der 4. Dekade)	Epitheliale Proliferation mit Schmelzorgandifferenzierung
	Etwas häufiger bei Männern	Gangartige Strukturen, kribriforme Architektur
		Keine *BRAF-*p.V600E-Mutationen
*Floride familiäre zementoossäre Dysplasie*	Früheres Auftreten als andere Subtypen, z. T. bereits bei Zahndurchbruch	Befall aller 4 Quadranten, i. d. R. deutlich expansiv
	Weniger starke Geschlechter- und Hautfarben-Assoziation als bei den anderen Subtypen	Selten hereditär (Case Reports)
*Segmentale odontomaxilläre Dysplasie*	1. bis 2. Dekade	Gehört zu den sog. Overgrowth-Syndromen
	Etwas häufiger bei Männern	Segmentale und einseitige Vergrößerung der Maxilla und der angrenzenden Weichteile
		Durchbruchstörungen und verschiedene Zahnanomalien
*Rhabdomyosarkom mit TFCP2-Rearrangement*	Junge Erwachsene (mittleres Alter 25 Jahre)	High-grade-Morphologie, teils spindelig, teils epitheloid
	Keine Geschlechterpräferenz	MYOD1- (und Myogenin‑) Expression
		*TFCP2*-Rearrangement und typischer Immunphänotyp mit Koexpression von AE1/E3 und ALK

## Odontogene Zysten und Tumoren

Nachdem in der dritten Auflage der WHO-Klassifikation die *odontogenen Zysten* nicht aufgeführt waren, da es sich in der Mehrzahl der Fälle nicht um eigentliche Tumoren handelt, sind die Läsionen in allen anderen Klassifikationen und nun auch in der 5. Auflage berücksichtigt. Ein Hauptargument besteht darin, dass die Zysten häufig in die Differenzialdiagnose anderer (zystischer) odontogener Tumoren einbezogen werden müssen. Bei einigen Zysten (z. B. odontogene Keratozyste, kalzifizierende odontogene Zyste) wurden inzwischen typische Mutationen nachgewiesen. Den Editor:innen der WHO-Klassifikationen reichen diese Veränderungen aber nicht aus, um diese Zysten als echte Neoplasien zu interpretieren, wobei dieses Konzept unter Expert:innen durchaus kontrovers diskutiert wird.

Die Gruppe der *odontogenen Zysten* führt als neuen Subtyp lediglich die *postoperative Flimmerepithelzyste* („post-surgical ciliated cyst“) auf. Diese Läsion ist bereits seit längerem bekannt und wird durch die traumatische/chirurgische Implantation von respiratorischem Epithel in den Kieferknochen verursacht. Es handelt sich um einen seltenen Befund, der meistens in der 5. oder 6. Dekade beobachtet wird, oft in der posterioren Maxilla [4]. Histologisch zeigt sich eine von respiratorischem Flimmerepithel ausgekleidete Zyste, die metaplastisches Plattenepithel enthalten und/oder sekundär entzündlich überlagert sein kann. Ätiologisch handelt es sich um chirurgisch transloziertes Sinusepithel, aus dem sich die Zysten ableiten. Differenzialdiagnostisch müssen u. a. glanduläre odontogene und follikuläre Zysten abgegrenzt werden. Wegweisend ist in der Regel die Anamnese, wobei ein ursächlicher Eingriff Jahre bis Jahrzehnte zurückliegen kann. Die Behandlung erfolgt durch Enukleation, Rezidive sind selten [22].

Die Definitionskriterien der *kalzifizierenden odontogenen Zyste* (KOZ) wurden geringfügig geändert, sodass für die Diagnose formal nur noch die charakteristischen Geisterzellen erforderlich sind. Neuere Studien konnten in mehr als 90 % der Fälle *CTNNB1*-Mutationen nachweisen [35]. Interessanterweise wurden *CTNNB1*-Mutationen auch in zahlreichen anderen Kopf-Hals-Tumoren mit Geisterzellen (oder ähnlichen Veränderungen) gefunden, sowohl in nichtodontogenen Tumoren (z. B. adamantinomatöse Kraniopharyngiome und Pilomatrixome) als auch in odontogenen Tumoren (dentinogene Geisterzelltumoren und odontogene Geisterzellkarzinome) [12].

In der Kategorie *odontogene Tumoren* gibt es nur wenige konzeptionelle Änderungen. Der Begriff „solides/multizystisches Ameloblastoms“ wurde durch „konventionelles Ameloblastom“ ersetzt. Weiterhin werden eine Reihe von histologischen Subtypen unterschieden (follikulär, plexiform, acanthomatös, granularzellig, basaloid und desmoplastisch). Unizystische (inkl. intraluminaler, luminaler und muraler Variante), periphere und metastasierende Ameloblastome werden als eigene Subtypen behandelt.

Das *adenoide Ameloblastom* (AA) wird erstmals als neuer Tumortyp aufgeführt. In der Literatur wurden bislang etwa 40 Fälle beschrieben, meist in der vierten Lebensdekade (Altersspanne 25–52 Jahre) [15]. Klinisch stellen sich die Patient:innen typischerweise mit einer schmerzlosen Schwellung des Unterkiefers vor, gelegentlich begleitet von Schmerzen und Parästhesien [2]. Morphologisch zeigen AA Epithelformationen mit Schmelzorgandifferenzierung, wobei typischerweise kribriforme, wirbelige und teils gangartige Strukturen ausgebildet werden, die Schleim enthalten können. Die basalen Zellen können mehrschichtig sein und in etwa zwei Dritteln der Fälle tritt zudem eine Bildung von aberrantem Dentin/Dentinoid auf (konventionelle Ameloblastome bilden keine Hartsubstanz). Der Ki-67-Proliferationsindex ist in der Regel hoch (bis zu 30 %) und korreliert mit dem lokal aggressiven Verhalten und einer relativ hohen Rezidivrate (bis zu 70 %) [15]. Differenzialdiagnostisch müssen adenomatoide odontogene Tumoren, klarzellige odontogene Karzinome (typischerweise mit *EWSR1* Rerrangements) sowie odontogene Karzinome mit Dentinoidbildung abgegrenzt werden, wobei die Kriterien insbesondere zu letztgenanntem Tumor nicht ganz scharf sind. Typischerweise sind beim AA pathogene Hotspotmutationen im *KRAS*-Gen nachweisbar, die bei den anderen Ameloblastom Subtypen selten sind. *BRAF*-p.V600E-Mutationen sind bisher nicht beschrieben [15].

Beim *kalzifizierenden epithelialen odontogenen Tumor* (KEOT) werden in der neuen Klassifikation 3 histopathologische Subtypen unterschieden: klarzellig, zystisch/mikrozystisch und nichtkalzifizierend/Langerhanszell-reich, wobei die Unterscheidung zwischen letztgenanntem Subtyp und dem amyloidreichen Subtyp des *odontogenen Fibroms* schwierig ist und unscharf bleibt. Die Neuinterpretation des ameloblastischen Fibrodentinoms und/-odontoms als frühes Stadium komplexer *Odontome* war bereits in der 4. Auflage der Klassifikation eine umstrittene Änderung und wird in der neuen Klassifikation beibehalten. Weiterhin wird jedoch betont, dass obwohl die überwiegende Mehrheit der Tumoren, die früher als ameloblastische Fibrodentinome und/oder Odontome bezeichnet wurden, Frühstadien von Odontomen sind und somit wahrscheinlich Hamartome darstelle, in Ausnahmefällen aber auch Läsionen mit stärker expansivem und nicht selbstlimitiertem Wachstum vorkommen, die wahrscheinlich echten Neoplasien entsprechen. Von solchen Tumoren dürften sich dann auch die seltenen ameloblastischen Fibrodentino- oder Fibroodontosarkome ableiten. Das *odontogene Myxom* mit erhöhtem Anteil an Kollagenfasern im Hintergrund wurde in der Klassifikation 2017 als Myxofibrom und wird neu nun als *Fibromyxom* bezeichnet. Die molekulare Pathogenese dieser Tumoren ist weiterhin unklar, Genpanelanalysen konnten keine Mutationen in typischen onkogenen Treibergenen nachweisen [27]. Eine Studie wies lediglich eine Überexpression von ERK1/2 nach und interpretierte diese als Anhaltspunkt für eine Aktivierung des MAP-Kinase-Signalwegs [25].

Bereits seit der 4. Auflage der WHO-Klassifikation wird das *zementoossifizierende Fibrom (ZOF)* als odontogener Tumor eingeordnet. Als Konsequenz dürfen nur solche Läsionen als ZOF klassifiziert werden, die sich in den zahntragenden Kieferabschnitten entwickeln, sodass eine Reihe von extragnathischen Tumoren mit ähnlicher Morphologie definitionsgemäß nicht klassifizierbar sind (ossifizierende Fibrome vom nichtodontogenen Typ, vgl. unten). Während die ZOF in der vierten Auflage noch zusammen mit den juvenilen trabekulären (JTOF) und psammomatoiden ossifizierenden Fibromen (POF) zusammen abgehandelt wurden, werden sie nun bei den gutartigen mesenchymalen odontogenen Tumoren aufgeführt (vgl. auch Abschn. „Zementoossifizierendes Fibrom (ZOF)“).

## Riesenzellhaltige Läsionen und Knochenzysten

Vor wenigen Jahren wurde nachgewiesen, dass zentralen *Riesenzellgranulomen* aktivierende Hotspotmutationen in den *KRAS-, FGFR1-* und *TRPV4-*Genen zugrunde liegen [11]. Auch periphere Riesenzellgranulome zeigen trotz ihres limitierten Wachstumspotenzials und gelegentlich spontaner Rückbildungen die gleichen genetischen Veränderungen [11]. In Übereinstimmung mit der Klassifikation von Knochen- und Weichteiltumoren wird der Begriff der sekundären aneurysmatischen Knochenzyste (AKZ) nicht mehr empfohlen und soll durch AKZ-artige Veränderungen oder zystische hämorrhagische Degeneration ersetzt werden. Solitäre Knochenzysten im peripheren Skelett weisen gemäß neuerer Untersuchungen Fusionstranskripte unter Beteiligung des *NFATC2*-Gens auf (typischerweise mit *EWSR1* und *FUS*) [26]. Im Kiefer ist diese Beobachtung noch nicht bestätigt worden.

## Fibroossäre Läsionen des Kiefers

Die fibroossären Läsionen (FOL) des Kiefers bilden eine Gruppe von Krankheitsbildern, die zahlreiche histologische Gemeinsamkeiten aufweisen, sich aber im klinischem Verhalten und radiologischen Charakteristika unterscheiden [21]. Angesichts der ähnlichen mikroskopischen Merkmale ist ein systematisches Vorgehen bei der Beurteilung unter Berücksichtigung des klinisch-radiologischen Kontexts von entscheidender Bedeutung, um Fehlinterpretationen zu vermeiden. Die neue Klassifikation führt erstmals den Subtyp der floriden familiären zementoossären Dysplasie (FFZOD) ein, der u. a. durch eine frühe Manifestation und teils ausgeprägte Expansion v. a. der vorderen Mandibula gekennzeichnet ist. Die Läsion muss vom äußerst seltenen, familiären gigantiformen Zementom (FGZ) unterschieden werden. Obwohl nicht neu und in der Literatur bereits ausführlich dokumentiert, wurde auch die segmentale odontomaxilläre Dysplasie (SOD) zum ersten Mal in die Klassifikation aufgenommen. Obwohl das ZOF neu in die Kategorie der mesenchymalen odontogenen Tumoren eingeordnet wurde, gehört es zum morphologischen Spektrum der fibroossären Läsionen und wird daher in diesem Kapitel behandelt.

### Psammomatoides ossifizierendes Fibrom (POF)

**Figure 1:**
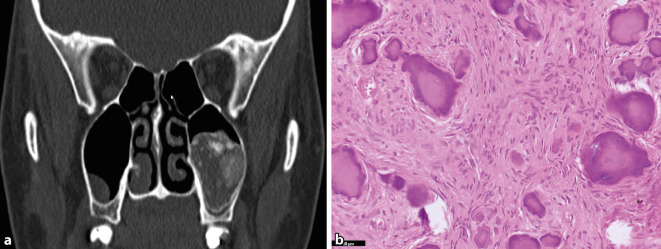

Das POF ist eine gutartige fibroossäre Läsion des kraniofazialen Skeletts. POF entstehen mehrheitlich in den Kiefer- und Schädelknochen, wobei etwa 90 % der Fälle von den Nasennebenhöhlen und der Orbita ausgehen [9]. Klinisch weisen die Patient:innen häufig eine asymptomatische, schnell wachsende Raumforderung auf (Abb. 1a). Im Gegensatz zum JTOF, tritt das POF eher bei Patient:innen in der zweiten und vierten Lebensdekade auf. Daher wird in der neuen WHO-Klassifikation der zuvor verwendete Begriff „juvenil“ für das POF aufgegeben. Histomorphologisch zeigt sich ein hyperzelluläres Spindelzellstroma ohne Atypien mit multiplen, zellarmen und sphärisch konfigurierten Matrixabscheidungen, die an Psammomkörperchen erinnern (Abb. 1b). Die Matrix dominiert häufig das histologische Bild und erscheint monomorph, Geflechtknochentrabekel kommen nur selten vor. Wenngleich histologisch Überschneidungen mit anderen fibroossären Kieferläsionen vorkommen, ist der Kontext aus vorwiegend extragnathischer Lokalisation, scharfer Begrenzung und Alter sehr charakteristisch. Auf genetischer Ebene sind reziproke Translokationen mit Bruchpunkten an Xq26 und 2q33 in 3 Fälle beschrieben [29]. Interessanterweise ließ sich dieser Befund in einer aktuellen Studie in 7 von 12 POF bestätigen, wobei das *SATB2*-Gen auf 2q33 als Partner verschiedener Fusionstranskripte nachgewiesen wurde (Cleven et al., *Modern Pathology*, accepted). Die Rezidivrate wird nach chirurgischer Exzision mit 30–56 % angegeben [28].

### Juveniles trabekuläres ossifizierendes Fibrom (JTOF)

**Figure 2:**
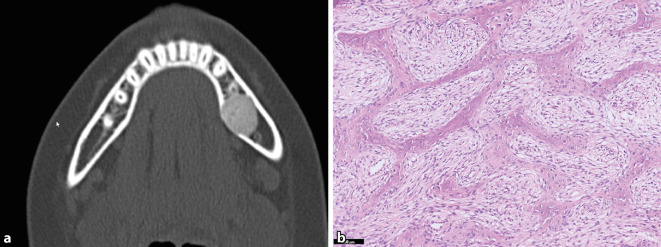

Das JTOF ist ein gutartiger fibroossärer Tumor, der vor allem im Kindesalter auftritt (Durchschnittsalter 11,3 Jahre) und mit nahezu gleicher Häufigkeit Ober- und Unterkiefer befällt [5]. Extragnatische JTOF sind in der Literatur vereinzelt beschrieben, es wird aber kontrovers diskutiert, ob diese Läsionen tatsächlich zum gleichen Spektrum gehören wie die JTOF des Kiefers. Klinisch präsentieren sich die Patient:innen mit schmerzlosen Raumforderungen, die sich radiologisch für gewöhnlich als scharf umschriebene und expansive Osteolysen darstellen (Abb. 2a). Histologisch findet sich ein zellreiches, monomorphes und fibroblastär imponierendes Spindelzellstroma mit unreifen trabekulären Geflechtknochenformationen, die fließend aus dem kollagenisierten Hintergrund hervorzugehen scheinen (Abb. 2b). Das eigentliche Ausmaß der Knochenbildung ist in frühen Läsionen oft nur in der Bindegewebsfärbung gut nachvollziehbar. Mit der Zeit reift die Matrix zu soliden Trabekeln aus, die miteinander anastomosieren. Immer wieder finden sich auch resorptionsaktive Osteoklasten, teilweise in umschriebenen Aggregaten. Der Kontext aus Alter, Bildgebung und unreifer Morphologie ist sehr typisch, sodass andere FOL normalerweise leicht abgegrenzt werden können. Die unreife Matrixbildung könnte an ein Osteosarkom erinnern, für das die Patient:innen aber in der Regel zu jung sind und für das überzeugende Atypien fehlen. Auch die Bildgebung ist nicht aggressiv genug für eine solche Differenzialdiagnose. Die molekulargenetische Pathogenese von JTOF ist unbekannt, maligne Transformationen kommen nicht vor [31]. Rezidive werden in ca. 20 % der Fälle beschrieben. Bei der Resektion sollte so konservativ wie möglich vorgegangen werden.

### Fibröse Dysplasie (FD)

**Figure 3:**
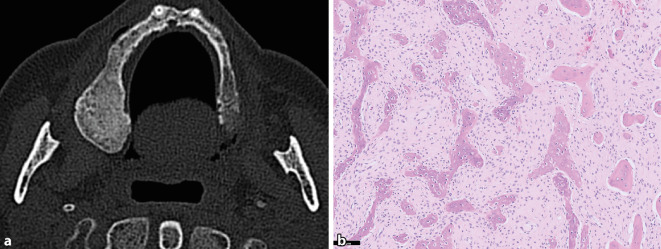

Die WHO-Klassifikation für Kopf-Hals-Tumoren definiert die FD als eine genetisch bedingte Störung des Knochenwachstums, die sich monostotisch oder polyostotisch manifestieren kann. Beim McCune-Albright-Syndrom tritt die FD zusammen mit endokrinen Störungen und Café-au-lait-Flecken auf [8]. Ursächlich sind postzygotische und aktivierenden Mutationen im *GNAS*-Gen, die nur bei diesem Krankheitsbild vorkommen und für die differenzialdiagnostische Abgrenzung gegenüber anderen FOL verwendet werden können [25]. Im Schädelbereich kann die FD kontinuierlich mehrere Schädelknochen miteinbeziehen, was ebenfalls als sehr spezifisch für diese Erkrankung gilt und im peripheren Skelett so nicht vorkommt. Klinisch präsentieren sich Patient:innen mit FD häufig mit einer schmerzlosen Schwellung, die gelegentlich auch entstellende Ausmaße annehmen kann. Im Kiefer tritt die FD häufiger im Oberkiefer als im Unterkiefer auf und ist in erster Linie eine Erkrankung des wachsenden Skeletts und damit von Kindern und Jugendlichen [18]. Das radiologische Erscheinungsbild variiert je nach Entwicklungsstadium und Alter des Patient:innen. Frühe Läsionen sind in der Regel stärker lytisch, nehmen über die Zeit aber einen homogenen Milchglasaspekt an (häufig verbleiben aber gemischt lytisch-sklerotische Anteile; Abb. 3a). Histomorphologisch erkennt man 2 Komponenten: ein fibröser Anteil bestehend aus reif imponierenden und fibroblastischen Spindelzellen, die an Zellularität über die Zeit abnehmen, und eine unreife Knochenbildung, oft mit einer auffallend kurvilineären Architektur der Geflechtknochenbälkchen, die in der Literatur mit chinesischen Schriftzeichen verglichen werden (Abb. 3b). In der Regel fehlen Osteoblastensäume auf der neugebildeten Matrix und in der Bindegewebsfärbung (z. B. Van-Gieson-Färbung) strahlen Sharpey-Fasern radiär von der Matrix in das umgebende Stroma aus. Mit der Zeit kann der läsionale Knochen ausreifen und mit dem umgebenden Knochen fusionieren, sodass es zum Teil schwierig sein kann, läsionalen und ortsständigen Knochen zu unterscheiden. Obwohl charakteristisch, ist das histologische Bild nicht völlig spezifisch. Die Differenzialdiagnose schließt andere fibroossäre Läsionen des Kiefers ein, z. B. ZOD und ZOF. Auch niedriggradige zentrale Osteosarkome können ähnlich aussehen wie eine FD, sind im Kiefer aber selten, zeigen eine aggressivere Bildgebung und weisen keine *GNAS*-Mutationen auf. Eine Untersuchung des *MDM2*-Gens kann in dieser Situation hilfreich sein, da etwa 25–30 % der zentralen low-grade Osteosarkome Amplifikationen aufweisen. FD sind gutartig, wobei es in seltenen Fällen zu maligner Transformation kommen kann [32].

Die FD galt lange Zeit als typisches Beispiel einer tumorähnlichen Läsion, wurde in der 5. Auflage der 2020 erschienen WHO-Klassifikation für Knochen- und Weichteiltumoren aber erstmalig als Neoplasie eingeordnet. In der Klassifikation der Kopf-Hals-Tumoren folgen die Editor:innen diesem Beispiel nicht und vermeiden den Begriff Neoplasie. Wenngleich es für beide Interpretationen Argumente gibt, wäre eine konsistentere Abstimmung der Klassifikationen untereinander hilfreich, speziell auch für so grundlegende Aspekte wie die Definition einer Neoplasie.

### Zementoossäre Dysplasie (ZOD)

**Figure 4:**
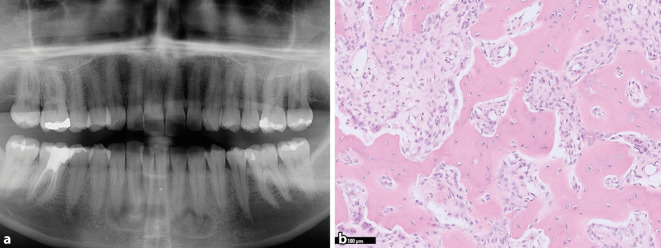

Die zementoossäre Dysplasie (ZOD) ist die häufigste gutartige fibroossäre Läsion des Kiefers [21]. Sie tritt ausschließlich in den zahntragenden Abschnitten auf, häufiger im Unterkiefer und vorwiegend bei Frauen mittleren Alters mit dunkler Hautfarbe. In der neuen Klassifikation werden 4 ZOD-Subtypen unterschieden: periapikal (PZOD, multifokal im Bereich der Schneidezähne des Unterkiefers), fokal (FZOD, unifokal, überall im Kiefer, mit Ausnahme der unteren Schneidezähne), floride (FLZOD, multifokal, oft mit Beteiligung mehrerer Quadranten, expansiv) und familiär floride (FFZOD, familiär, frühes Auftreten, v. a. vorderer Unterkiefer, expansiv). Klinisch handelt es sich bei den meisten Formen um asymptomatische Zufallsbefunde, wobei die floriden Subtypen auch als Schwellungen klinisch apparent werden können [24]. Die Bildgebung ist für die Diagnose von entscheidender Bedeutung und klassische Präsentationen können auch ohne Biopsie zuverlässig diagnostiziert werden. Frühe Formen präsentieren sich als umschriebene periapikale Osteolysen, die über die Zeit (Jahre) von zentral nach peripher mineralisieren (Abb. 4a). Insbesondere beim periapikalen Subtyp kann es dabei zur Fusion einzelner Läsionen kommen. Ein expansives Wachstum kann bei allen Subtypen auftreten, ist bei periapikalen und fokalen Formen aber eher die Ausnahme. Alle ZOD sind selbstlimitierend. Histologisch variiert das Bild ebenfalls in Abhängigkeit des Reifestadiums. Initial dominiert das fibroblastäre Spindelzellstroma über unreife Geflechtknochentrabekel und zementikelartige Abscheidungen (Abb. 4b). Über die Zeit kommt es zur Ausbildung einer plumpen, scholligen und zellarmen Matrix, die sehr typisch ist und deren Form von einigen Autor:innen als ingwerwurzelähnlich beschrieben wurde [23]. ZOD wurden traditionell als nichtneoplastisch angesehen. Unsere Arbeitsgruppe fand aber kürzlich pathogene Hotspotmutationen in Genen des MAP-Kinase-Signalwegs in etwa einem Drittel der untersuchten Läsionen, sodass es sich möglicherweise doch um (selbstlimitierte) Neoplasien handelt [13]. Die Läsionen sind gutartig und maligne Transformationen kommen nicht vor. Auch eine spezifisch Therapie ist in der Regel nicht erforderlich, solange die Patient:innen asymptomatisch sind. Sekundäre Infektionen können aufgrund der schlechten Vaskularisierung klinisch schwierig zu therapieren sein, weshalb unnötige Eingriffe (auch diagnostische Biopsien) vermieden werden sollten.

In der neuen Klassifikation wurde erstmals die FFZOD als neuer Subtyp eingeführt. Im Gegensatz zur FZOD, tritt sie bereits früher auf, zum Teil bereits zum Zeitbruch des Zahndurchbruchs und es kann v. a. im Bereich des vorderen Unterkiefers zu einem deutlichen expansiven Wachstum kommen [20]. Die Unterscheidung zwischen FFZOD und FGZ kann klinisch und histologisch schwierig sein. Patient:innen mit FGZ zeigen allerdings häufig einen diffuseren Befall mehrerer Quadranten und einen noch früheren Krankheitsbeginn.

### Segmentale odontomaxilläre Dysplasie (SOD)

Auch die segmentale odontomaxilläre Dysplasie (SOD) wird erstmals in der Gruppe der fibroossären Läsionen aufgeführt [30]. Die SOD ist definiert als eine einseitige Entwicklungsstörung mit segmentaler Vergrößerung des Oberkiefers, dentoossären Anomalien und gelegentlichen kutanen Manifestationen. Klinisch zeigt sich eine schmerzlose, oft prämolar/molar betonte Expansion des Oberkiefers, in der Regel in den ersten beiden Dekaden. Zu den dentalen und kutanen Anomalien gehören fehlende permanente Zähne sowie Hypertrichose und Hyper- bzw. Hypopigmentierung im Gesichtsbereich. Die Ursachen der SOD sind nicht bekannt. Pränatale Traumata, endokrine Anomalien und bakterielle/virale Infektionen werden als mögliche Auslöser diskutiert. Histologisch zeigt sich eine zellarme Fibrose mit gelegentlichen dystrophen Verkalkungen und inerte Knochenformationen mit irregulärem Kittlinienmuster, aber ohne wesentliche osteoblastäre oder osteoklastäre Aktivität. Wurzelresorptionen von Zähnen mit dysplastischem Dentin und Pulpafibrose wurden ebenfalls beschrieben. Differenzialdiagnostisch muss in erster Linie eine FD ausgeschlossen werden. Der Verlauf der SOD ist gutartig, eine modellierende Abtragung kann allerdings aus kosmetischen und/oder funktionellen Gründen erforderlich sein. Interessanterweise wurden bei betroffenen Patient:innen Low-level-Mosaik-Variationen im *PIK3CA*-Gen gefunden, die auf einen Zusammenhang mit anderen *PIK3CA*-assoziierten Overgrowth-Syndromen hinweisen [10].

### Zementoossifizierendes Fibrom (ZOF)

**Figure 5:**
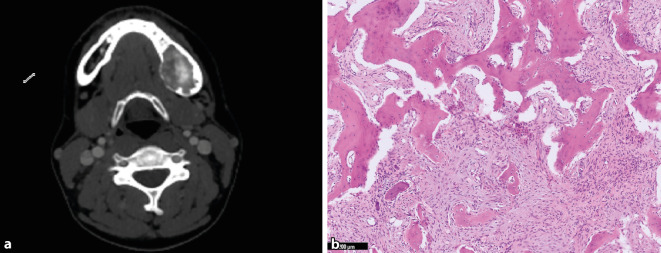

Das ZOF wurde bereits in der 4. Auflage der WHO-Klassifikation von 2017 als echter odontogener Tumor definiert, damals aber noch unter den fibroossären Läsionen beschrieben. In der aktuellen Klassifikation ist das ZOF unter den gutartigen mesenchymalen odontogenen Tumoren aufgeführt und somit noch stärker von anderen fibroossären Läsionen abgegrenzt. Ob diese Unterscheidung sinnvoll ist, bleibt abzuwarten, auch unter Berücksichtigung der neuen molekularen Befunde bei den zementoossären Dysplasien, die somit ebenfalls in diese Kategorie gehören. Definitionsgemäß kommen ZOD nur in den zahntragenden Kieferabschnitten vor, bevorzugt im Unterkiefer im (Prä‑)Molarenbereich [14]. Im Gegensatz zu POF und JTOF präsentieren sich ZOF-Patient:innen oft mit einer eher langsam fortschreitenden Raumforderung, die radiologisch scharf umschrieben und expansiv ist (Abb. 5a). Morphologisch besteht das ZOF aus einer Mischung von monomorphen fibroblastären Spindelzellen sowie unreifen Knochentrabekeln und zementikelartiger Matrix (Abb. 5b). Säume kubischer Osteoblasten auf der Oberfläche der läsionalen Trabekel sind ein typisches, aber keineswegs spezifisches Merkmal. Die Zellularität des Stromas variiert, signifikante zelluläre Atypien kommen nicht vor. Typischerweise ist das ZOF von einer dünnen Bindegewebsschicht umgeben, welche eine Verschmelzung mit dem ortständigen Knochen verhindert (bei den ZOD fusioniert die läsionale Matrix mit dem Knochen). Die molekulare Pathogenese ist bislang unklar. Selten können ZOF in Zusammenhang mit inaktivierenden Mutationen im Tumorsuppressorgen *CDC73* (HRPT2) als Teil eines Hyperparathyreoidismus-Kiefertumor-Syndroms vorkommen, wobei es sich praktisch um die einzige Ausnahme eines multifokalen Auftretens handelt (sonst immer unifokal) [19]. ZOF wurden auch selten in Zusammenhang mit einer GDD mit Mutationen im *ANO5*-Gen beschrieben [1]. Rezente Studien an sporadischen ZOF konnten mithilfe von Genpanelsequenzierungen keine Mutationen in onkogenen Treibergenen aufzeigen [24]. Tabareau-Delalande et al. hatten in einer Serie von ZOF mittels qPCR *MDM2*-Amplifikationen nachgewiesen, die aber nicht mit einer Überexpression assoziiert waren und nicht mittels Fluoreszenz-in-situ-Hybridisierung (FISH) oder genomweiter Kopienzahlanalysen verifiziert wurden [33]. Die Autoren diskutieren dennoch ZOF als potenzielle Vorläuferläsion von zentralen low-grade Osteosarkomen, wofür unseres Erachtens die Datenlage nicht ausreicht. Auch in der Literatur wurden bislang keine weiteren Fälle von ZOF mit *MDM2*-Amplifikation oder gar maligner Transformation beschrieben. Wir gehen daher weiterhin davon aus, dass es sich um benigne Tumoren handelt. Die Prognose ist gut und eine Kürettage oder Enukleation ist in der Regel ausreichend [14].

### Ossifizierendes Fibrom vom nichtodontogenen Typ

Unsere Arbeitsgruppe hat vor Kurzem eine Serie von fibroossären Läsionen beschrieben, die sowohl im Unterkiefer (unterhalb des Nervenkanals) als auch extragnathisch, v. a. im Bereich des Os frontale, auftreten und für die es seit der Definition des ZOF als odontogenen Tumor keine Kategorie in der WHO-Klassifikation gibt [3]. Es handelt sich um expansive Raumforderungen mit spindeliger Stromazellkomponente ohne Atypien und einer unreifen, häufig auffallend zellarmen Matrixbildung. Die zelluläre Proliferation war niedrig und es handelte sich ausschließlich um asymptomatische Zufallsbefunde mit klinisch indolentem Verlauf. Interessanterweise waren mehr Männer betroffen (14/20), das Durchschnittsalter betrug 44 Jahre (Range 27–74 Jahre). Wir haben unsere Serie mit dem zuständigen Editor des ZOF-Kapitels, Prof. Edward Odell, diskutiert, der die Studie im Kapitel auch zitiert. Als provisorischen Namen haben wir das ossifizierende Fibrom vom nichtodontogenen Typ vorgeschlagen.

## Benigne Knochen- und Knorpeltumoren

In dieser Kategorie gibt es, abgesehen von der bereits beschriebenen Neueinstufung des ZOF als odontogene Neoplasie, keine wesentlichen Änderungen. Auf genetischer Ebene konnten wir beim Zementoblastom erstmals *FOS*-Rearrangements beschreiben, die auf eine Verwandtschaft mit den morphologisch ähnlichen Osteoidosteomen und Osteoblastomen des peripheren Skeletts hinweisen [16].

## Maligne Knochen- und Knorpeltumoren

**Figure 6:**
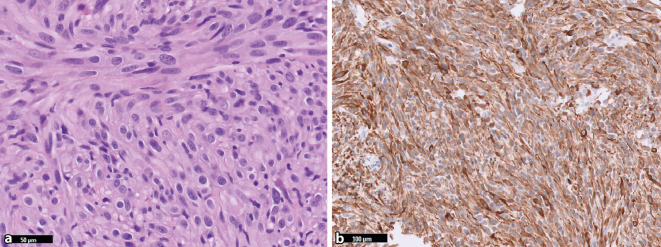

In der aktuellen Klassifikation werden die im Kiefer‑/Gesichtsbereich seltenen Chondrosarkome mit ihren Subtypen (konventionell, periostal, dedifferenziert und klarzellig) als „Chondrosarkom-Tumorfamilie“ zusammengefasst und nur die hier häufiger vorkommenden mesenchymalen Chondrosarkome separat aufgeführt. Ein neuer und erstmalig aufgeführter Knochentumor ist das Rhabdomyosarkom mit *TFCP2*-Rearrangement, bei dem das *TFCP2* Gen mit *EWSR1* oder *FUS* fusioniert [17]. Es handelt sich um ein high-grade Sarkom, das hauptsächlich in den Kieferknochen junger Erwachsener vorkommt (Durchschnittsalter 25 Jahre). Radiologisch sind die Tumoren in der Regel unscharf begrenzt und infiltrieren das umliegende Weichgewebe. Histomorphologisch zeigt sich ein biphasisches Muster aus spindeligen und epitheloiden Proliferaten (Abb. 6a), wobei in seltenen Fällen auch nur eine dieser Komponenten vorhanden sein kann. Die Kerne der Tumorzellen sind groß, monoton und weisen deutlich sichtbare Nukleolen auf. Nekrosen und Mitosefiguren sind häufig. Die Tumorzellen sind positiv für Zytokeratine (AE1/AE3; Abb. 6b), Desmin und MyoD in einem Teil der Fälle auch für Myogenin und ALK. Aufgrund der epithelioiden Morphologie und der Positivität für Zytokeratine kann der Befund als Karzinom fehlinterpretiert werden. Bei ungewöhnlicher Morphologie und Alter sollten daher Desmin und MyoD1 großzügig eingesetzt werden, um diese Differenzialdiagnose nicht zu verpassen. Beweisend ist der Nachweis des Fusionstranskripts, alternativ und schneller kann auch eine FISH-Untersuchung die wegweisende Aberration nachweisen [6, 17]. Die Tumoren weisen neben der Translokation zudem komplexe genomische Profile mit homozygoten *CDKN2A*-Deletionen in der Mehrheit der Fälle auf. Andere genetische Veränderungen umfassen *MDM2-*Amplifikationen, Mutationen von *TP53* sowie Hochregulierungen von *ALK* und *TERT *[7, 17, 34]. Die Prognose ist schlecht. Die meisten Patienten weisen eine lokal fortgeschrittene Erkrankung auf und in mehr als der Hälfte der Fälle entwickeln sich Fernmetastasen, entweder zum Zeitpunkt der Diagnose oder später im Verlauf der Erkrankung [17].

## Fazit für die Praxis

- Die 5. Auflage der WHO-Klassifikation für Kopf-Hals-Tumoren enthält nur wenige konzeptionelle Änderungen im Vergleich zur Vorversion. Alle Kapitel wurden aber aktualisiert und neu verfasst mit einem umfassenden Update der molekulargenetischen Charakteristika der verschiedenen Kieferläsionen.
- Bei den diagnostischen Kriterien wird nun, analog zu den anderen WHO-Klassifikationen, in „essential“ und „desirable“ unterschieden.
- Zu den erstmalig aufgeführten Entitäten in der Gruppe der odontogenen Tumoren und Kiefertumoren gehören die postoperative Flimmerepithelzyste, das adenoide Ameloblastom, die segmentale odontomaxilläre Dysplasie, die floride familiäre zementoossäre Dysplasie und das Rhabdomyosarkom mit *TFCP2*-Rearrangement.
- Die Berücksichtigung des klinisch-radiologischen Kontexts und damit eines interdisziplinären Ansatzes ist für die Diagnosestellung und Klassifikation der fibroossären Läsionen des Kiefers von entscheidender Bedeutung. Für die meisten Kieferläsionen sind auch weiterhin keine molekularen Zusatzuntersuchungen erforderlich.
